# Perioptic Cerebrospinal Fluid Dynamics in Idiopathic Intracranial Hypertension

**DOI:** 10.3389/fneur.2018.00506

**Published:** 2018-06-28

**Authors:** Achmed Pircher, Margherita Montali, Joachim Pircher, Jatta Berberat, Luca Remonda, Hanspeter E. Killer

**Affiliations:** ^1^Department of Ophthalmology, Cantonal Hospital, Aarau, Switzerland; ^2^Department of Ophthalmology, San Bassiano Hospital, Bassano del Grappa, Italy; ^3^Department of Cardiology, Ludwig-Maximilians-Universität München, Munich, Germany; ^4^Department of Neuroradiology, Cantonal Hospital, Aarau, Switzerland

**Keywords:** idiopathic intracranial hypertension, cerebrospinal fluid, optic nerve, optic canal, optic nerve sheath compartment

## Abstract

**Purpose:** To examine the cerebrospinal fluid (CSF) dynamics along the entire optic nerve in patients with idiopathic intracranial hypertension (IIH) and papilledema by computed tomographic (CT) cisternography.

**Methods:** Retrospective analysis of CT cisternographies in 16 patients with a history of IIH and papilledema (14 females and 2 males, mean age: 49 ± 16 years). Contrast loaded CSF (CLCSF) was measured in Hounsfield Units (HU) at three defined regions of interest (ROI) along the optic nerve (orbital optic nerve portion: bulbar and mid-orbital segment, intracranial optic nerve portion) and additionally in the basal cistern. The density measurements in ROI 1, ROI 2, and ROI 3 consist of measurements of the optic nerve complex: optic nerve sheath, CLCSF filled SAS and optic nerve tissue. As controls served a group of patients (mean age: 60 ± 19 years) without elevated intracranial pressure and without papilledema.

**Results:** In IIH patients the mean CLCSF density in the bulbar segment measured 65 ± 53 HU on the right and 63 ± 35 HU on the left side, in the mid-orbital segment 68 ± 37 HU right and 60 ± 21 HU left. In the intracranial optic nerve portion 303 ± 137 HU right and 323 ± 169 HU left and in the basal cistern 623 ± 188 HU. Within the optic nerve the difference of CLCSF density showed a highly statistical difference (*p* < 0.001) between the intracranial optic nerve portion and the mid-orbital segment. CLCSF density was statistically significantly (*p* < 0.001) reduced in both intraorbital optic nerve segments in patients with IIH compared to controls.

**Conclusions:** The current study demonstrates reduced CLCSF density within the orbital optic nerve segments in patients with IIH and papilledema compared to 12 controls without elevated intracranial pressure and without papilledema. Impaired CSF dynamics could be involved in the pathophysiology of optic nerve damage in PE in IIH.

## Introduction

Idiopathic intracranial hypertension (IIH) is a neurological disorder characterized by elevated intracranial pressure (ICP) in the absence of any known causative factor (mass lesion, inflammation, central venous thrombosis). The patients typically present with headache, visual disturbances (transient visual obscurations), tinnitus and diplopia. Mainly affected are obese women of childbearing age (1, 2). IIH can be self-limiting, relapsing or chronic. Normally patients with IIH develop papilledema, that if left untreated, may lead to permanent visual loss (3–6).

By definition papilledema refers to a swelling of the optic disc resulting from increased ICP. The development of papilledema is thought to depend on the transmission of elevated ICP via the subarachnoid space (SAS) to the lamina cribrosa with resulting damage to axonal transport (7). This theory assumes a free CSF circulation through the optic canal between the intracranial SAS and the orbital SAS of the optic nerve.

Papilledema is usually bilateral but highly asymmetric cases and even unilateral cases have been described (8–11). Further there are case reports of patients that presented with persisting papilledema after successful ICP lowering by shunt procedures (12, 13) and there are cases of IIH without papilledema (14–16).

The understanding of the pathophysiology in patients with IIH and persistent papilledema is of great importance as in these patients visual impairment progresses and can result in permanent visual loss (17, 18).

Although several studies (7, 19, 20) have demonstrated pressure induced damage of the axonal transport in the presence of papilledema, our knowledge about CSF dynamics along the entire optic nerve in papilledema and it's relationship to elevated ICP is poor.

In the current study the contrast loaded CSF (CLCSF) density was measured by computer tomographic (CT) assisted cisternography at three regions along the optic nerve (orbital optic nerve portion: bulbar segment and mid-orbital segment, intracranial optic nerve portion) and in the basal cistern in 16 patients with history of IIH and papilledema with visual impairment. A group of 12 patients without known history of elevated ICP and papilledema that underwent CT cisternography for various reasons served as controls.

This study should add to the understanding of the complex CSF dynamics in the SAS along the entire optic nerve in patients with IIH and papilledema.

## Materials and methods

This study was approved by the local ethical commission (Ethikkommission Nordwest- und Zentralschweiz) and follows the tenets of the Declaration of Helsinki. The informed consent from the subjects was checked before inclusion in this study.

### Patients with idiopathic intracranial hypertension

From 2005 to 2015 16 patients (32 optic nerves), 14 women and 2 men with an established diagnosis of IIH were admitted to our department for CT cysternography because of a therapy-resistant papilledema and progressive visual impairment (visual acuity and/or visual field). Mean age was 49 ± 16 years, 47 ± 15 years in women and 68 ± 15 years in men. All patients underwent a full neuro-opthalmological examination, magnetic resonance imaging (MRI) and lumbar puncture to establish the diagnosis. This was based on the updated modified Dandy criteria (21).

At initially presentation all patients had bilateral papilledema (no information about grade). The mean CSF opening pressure measured by lumbar puncture was 40.0 ± 12.7 cmH2O (in 5 patients the lumbar CSF pressure was documented from the performing doctor as >25 cmH2O) at time of diagnosis.

At time of cisternography in all patients both optic nerves were involved whereas in 8 patients papilledema was slightly asymmetrical and 2 patients showed atrophic optic discs. The average visual field mean deviation was 6.6 ± 8.5 dB on the right and 6.9 ± 7.9 dB on the left side using standard automated perimetry (SAP, Program G2 Octopus Haag-Streit, Switzerland) whereas in 5 patients only the Goldman visual field perimetry was used. The visual acuity using the logMAR chart was 0.3 ± 0.5 right and 0.2 ± 0.3 left. The mean body mass index (BMI) was 31 ± 5. The mean CSF opening pressure measured by lumbar puncture was 29.2 ± 8.6 cmH2O at time of cisternography (Table 1).

**Table 1:** Clinical parameters at time of cisternography of patients with Idiopathic Intracranial Hypertension (IIH).

**Patients**		**BMI**	**Visual Acuity**		**Visual Field**		**CSF-p**
***n***	**Age [years]**	**kg/m^2^**	**OD**	**OS**	**OD**	**OS**	**cmH2O**
			**logMAR**		**MD**		
1	60–65	36	0	0	3.9	4.3	21
2	26–30	37	0.1	0	2.8	0.7	18
3	75–80	25	1.3	0.1	25.2	25.9	24
4	56–60	38	0	0.1	1.4	9.2	26
5	20–25	24	0	0	19.4	13.7	29
6	56–60	32	0	0	0.9	0.3	34
7	20–25	33	0	0	2.1	2.7	45
8	46–50	36	0	0	2.9	2.2	34
9	46–50	33	0	1	–	–	29
10	40–45	33	0	0	5.3	7.2	25
11	36–40	35	0.1	0	–	–	28
12	60–65	30	1	0.5	–	–	23
13	56–60	29	0.3	0.2	–	–	50
14	56–60	24	0	0	2.4	2.3	34
15	30–35	27	1.3	1	–	–	27
16	60–65	27	0	0	12	12.2	21
AM ± SD	49 ± 16	31 ± 5	0.3 ± 0.5	0.2 ± 0.3	6.6 ± 8.5	6.9 ± 7.9	29.2 ± 8.6

*BMI, body mass index; MD, mean deviation; CSF-p, cerebrospinal fluid pressure; AM, arithmetic mean; SD, standard deviation; OD, oculus dexter (right optic nerve); OS, oculus sinister (left optic nerve)*.

Clinical symptoms were visual symptoms (blurry vision, transient visual obscuration, visual field impairment) (16 patients), headache (12 patients), and diplopia from VIth cranial nerve palsy (1 patient). At time of cisternography all patients were treated with oral acetozolamide (10 patients: 1,500 mg/day, 6 patients: 1,000 mg/day due to side effects, mean dose: 1,313 mg/day) and 2 patients had additionally undergone a shunt procedure. The duration of the therapy was variable depending on the duration of the disease. Radiological signs showed flattening of the posterior globe (16 patients), an “empty sella” (6 patients), and stenosis of the transverse venous sinus (3 patients).

The indication for CT cysternography was progressive visual impairment and persistent papilledema (14 patients) or optic nerve atrophy development (2 patients) despite weight loss attempts and maximal therapy with systemic acetazolamide or additionally performed shunt procedures. The mean time from diagnosis to performed CT cisternography was highly variable and varied between 23 years and 3 months, the mean was 5 ± 6 years.

During CT cisternography all patients were hospitalized in our department and underwent a full ophthalmological and neuro-ophthalmological examination including slit lamp-assisted biomicroscopy and visual field testing.

### Controls

As CT cisternography is an invasive and therefore potentially harmful technique, a healthy aged-matched control group is not available. We therefore included an inhomogeneous group of patients who underwent CT cisternography for a variety of indications. The same group was used as controls in a not yet published work about glaucoma.

From the database of the Department of Neuroradiology, Cantonal Hospital, Aarau, Switzerland, all patients who underwent CT cisternography for various diagnostic reasons in the period from 2010 to 2015 were used as the initial dataset. 12 patients (24 optic nerves), 9 women and 3 men without elevated intracranial pressure (<20cmH2O) and without papilledema were included and served as controls. Mean age was 60 ± 19 years, 61 ± 22 years in women and 55 ± 10 years in men. There was no significant difference (*p* = 0.13, unpaired *t*-test) in age between subjects in the IIH group and control group. Exclusion and inclusion criteria were verified in the medical records. Six of the controls underwent CT cisternography in order to exclude leakage of CSF, in 4 after endonasal sinus surgery because of chronic sinusitis and in 2 after a neurosurgery intervention (cyst excision). In the other 6 controls CT cisternography was performed because of neurological symptoms without cytologic or chemical abnormalities in CSF and without pathological findings in MRI or CT.

The mean CSF opening pressure measured by lumbar puncture was 13.0 ± 4cmH2O (in 8 patients the lumbar CSF-p was documented as <20cmH2O) at time of cisternography.

At the beginning of cisternography a lumbar puncture was performed in the lateral decubitus position and CSF pressure was measured. The patient‘s legs were straightened and the patient was asked to remain calm and not to speak during the measurements in order to avoid Valsalva manoeuvers. In all patients with IIH during lumbar puncture 10 ml of CSF was sampled for biochemical analysis and then 10 ml iopamidol (molecular weight 778 D, Iopamiro 300, Bracco, Milano, Italy) was injected intrathecally. The total CSF volume was therefore not altered. The patient was then turned to the prone position. The time between iopamidol injection and CT was 15 min and was the same in both patients and controls. All patients underwent CT cisternography at the same institution, by the same neuro-radiologist (L.R.).

A 64-detector scanner (Aquillion 64, Toshiba, Tokyo, Japan) providing 0.5 mmX32 section collimation was used. Scanning parameters were a 25 cm field of view with a 512 × 512 matrix and a soft tissue and a bone reconstruction algorithm were employed. The field of view included the foramen magnum and the nose.

Multiplanar reconstruction images (MPR) were obtained in the axial, coronal and sagittal planes with a 0.5 mm slice thickness. CT images were analyzed using the program VitreaCore (Vital Images, Inc., Minnetonka, MN, USA) on the Advantage Workstation 4.1 software (General Electric, Milwaukee, Wisconsin, USA).

**Figure 1:**
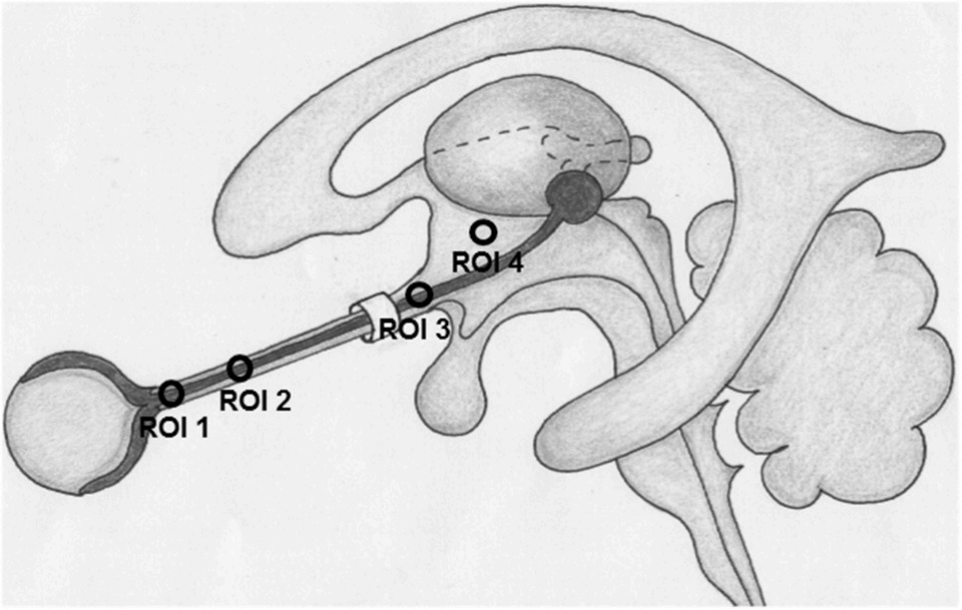
Region of interests (ROI) where contrast loaded cerebrospinal fluid (CLCSF) density measurements were performed. ROI 1: bulbar segment of the orbital portion, ROI 2: mid-orbital segment of the orbital portion, ROI 3: intracranial portion, ROI 4: basal cistern.

**Figure 2:**
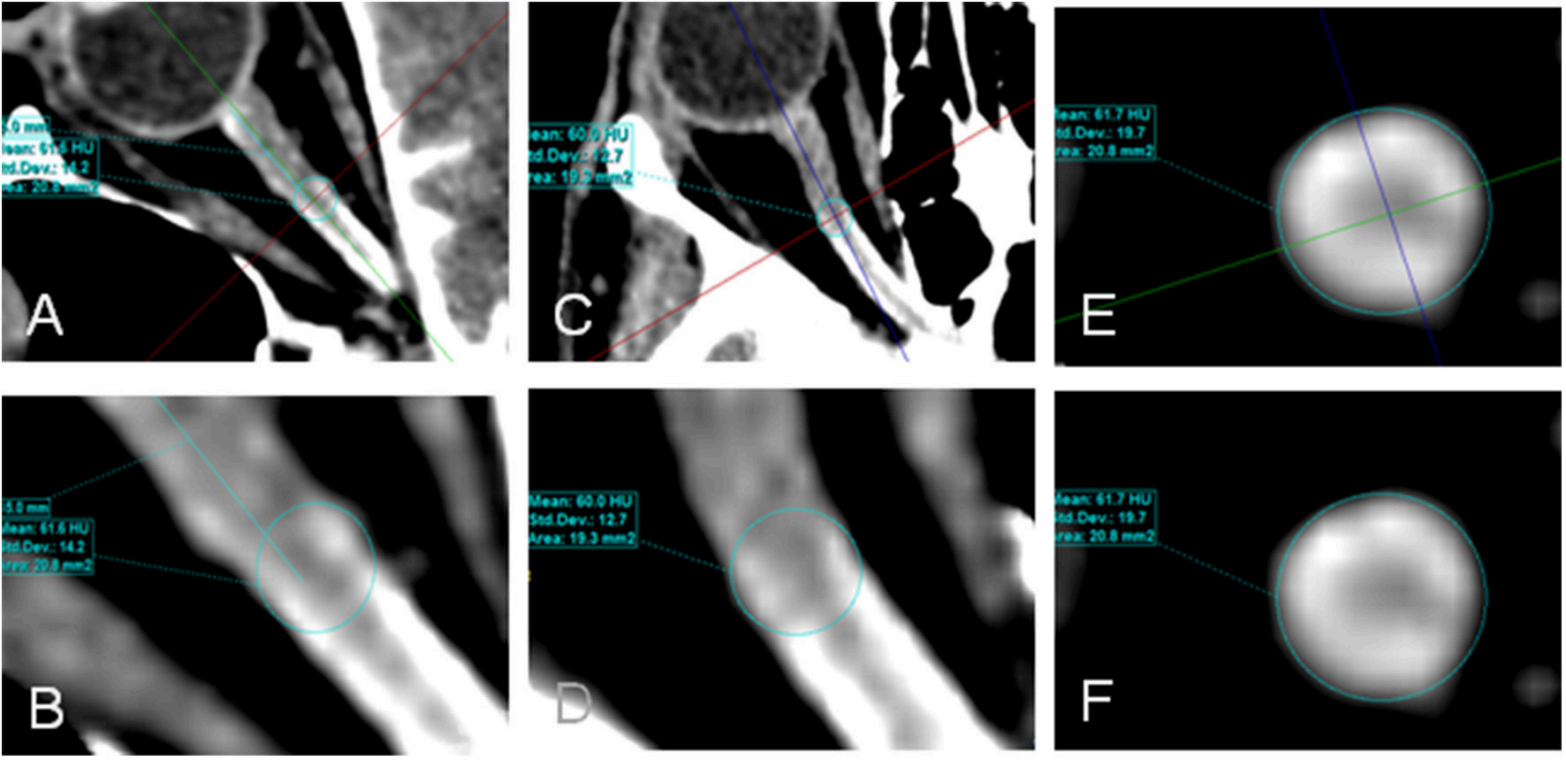
Contrast loaded cerebrospinal fluid (CLCSF) density measurements in the mid-orbital segment of the orbital portion (ROI 2) of a right optic nerve. Axial **(A,B)**, sagittal **(C,D)**, and coronal **(E,F)** sections were used.

CLCSF density was measured in Hounsfield units (HU) on CT images. Measurements of the contrast agent were performed at three defined regions along the optic nerve and in the basal cistern. Region of interest (ROI) 1 (bulbar segment) was defined as the area 1–4 mm in distance from the lamina cribrosa, ROI 2 (mid-orbital segment), as the area 13–16 mm in distance from lamina cribrosa and ROI 3 (intracranial portion) was defined as the area from the optic chiasm to the cranial opening of the OC. ROI 4 corresponded to the basal cistern (Figure 1). In ROI 1, ROI 2, and ROI 3 the HU measurements include also the optic nerve because it is technically not possible to measure the CSF filled SAS alone. The measurements in these ROIs are therefore the “optic nerve complex density” which consists of optic nerve sheath, CLCSF filled SAS and optic nerve tissue. The border of the optic nerve complex was defined by the diameter of the optic nerve sheath. Measurements were performed in axial, sagittal and coronal sections after the images have been formatted into a coronal plane perpendicular to the sagittal axis of the optic nerve, in ROI 4 only the axial section was used (Figure 2).

All CLCSF measurements were measured twice and reviewed by an experienced neuroradiologist blinded to the neuro-ophthalmological findings.

Statistical analysis was performed using SigmaPlot 10.0 (Systat Software, San Jose, CA, USA), Microsoft Excel 2010 (Microsoft Corporation, Redmond, WA, USA) for Windows statistical package. Data were analyzed using paired or unpaired Student *t*-test as appropriate to compare normally distributed variables, and Wilcoxon Signed Rank test or Mann-Whitney *U-*test as appropriate when normal distribution was not given. All data are expressed as mean and standard deviation or median and interquartile ranges respectively. Differences were considered significant when the error probability was *p* < 0.05.

## Results

### CT cisternography

#### Bulbar segment of orbital optic nerve portion: ROI 1 (1–4 mm distant from the lamina cribrosa)

**Figure 3:**
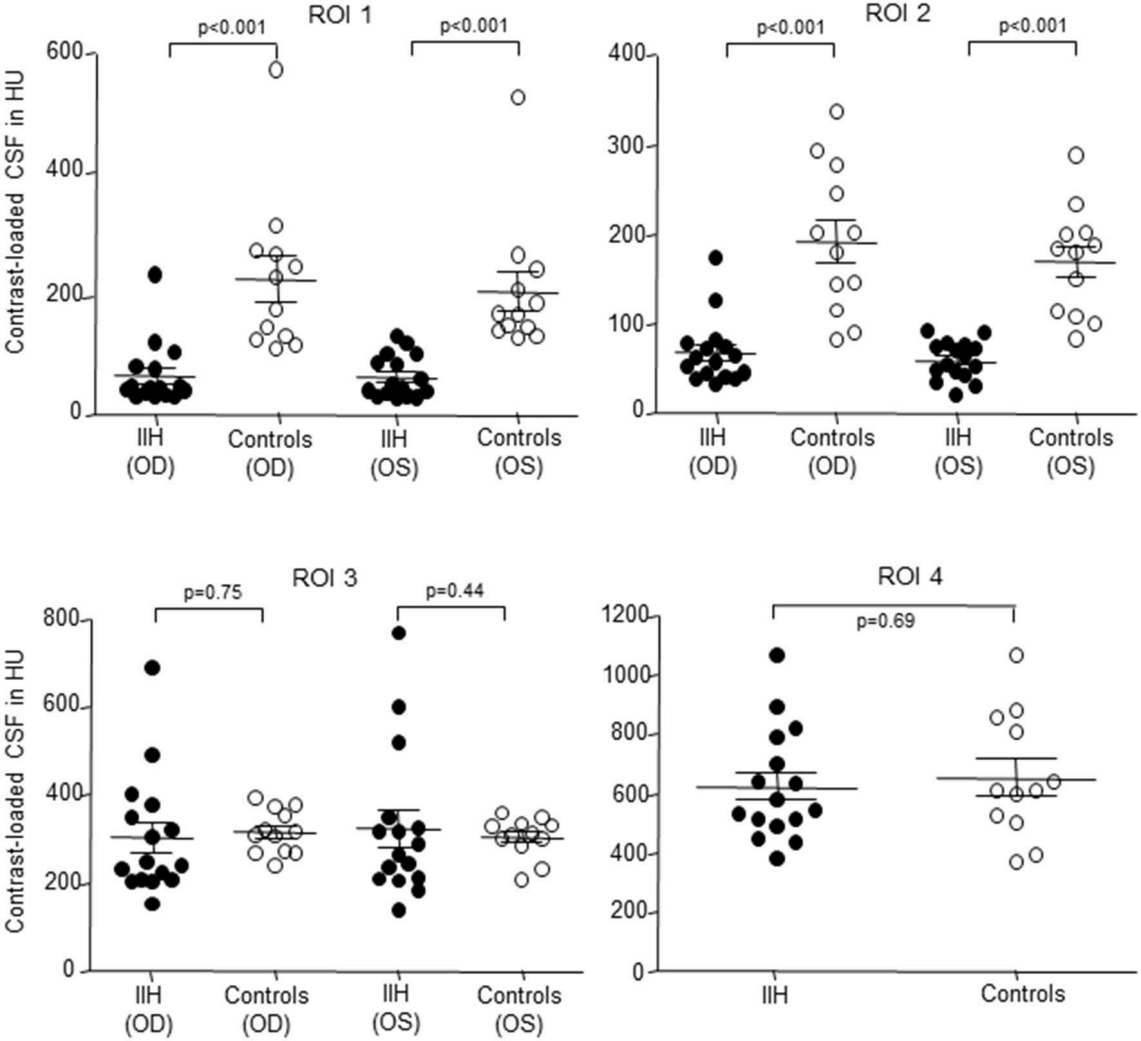
Scatterplot of contrast loaded cerebrospinal fluid (CLCSF) density measurements in Hounsfield Units (HU) in the bulbar- (ROI 1) and mid-orbital segment (ROI 2) of the optic nerve, the intracranial portion (ROI 3) of the optic nerve and in the basal cistern (ROI 4) in patients with idiopathic intracranial hypertension (IIH) and papilledema and controls, separated for right and left optic nerve. IIH patients (*n* = 16) and controls (*n* = 12). •, IIH patients; ◦, controls; OD, oculus dexter (right optic nerve); OS, oculus sinister (left optic nerve).

In IIH patients the mean density of CLCSF measured 65 ± 53 HU in the right optic nerve and 63 ± 35 HU in the left optic nerve. In controls the mean CLCSF density measured 226 ± 131 HU in the right optic nerve and 205 ± 111 HU in the left optic nerve (Tables 2, 3). The difference between IIH patients and controls showed statistical significance for the right and for the left side (right optic nerve: *p* < 0.001, left optic nerve: *p* < 0.001, Mann-Whitney *U*-test) (Figure 3).

**Table 2:** Measurements of contrast loaded cerebrospinal fluid (CLCSF) along the entire optic nerve in patients with Idiopathic Intracranial Hypertension (IIH).

**Patients**		**Basal cistern**	**Intracranial portion**		**Mid-orbital segment**		**Bulbar segment**	
		**[ROI 4]**	**[ROI 3]**		**[ROI 2]**		**[ROI 1]**	
***n***	**Age**		**OD**	**OS**	**OD**	**OS**	**OD**	**OS**
	**Years**	**HU**	**HU**		**HU**		**HU**	
1	60–65	542	203	205	62	47	43	39
2	26–30	701	231	287	127	78	233	118
3	75–80	1070	689	769	33	42	40	40
4	56–60	820	492	317	74	75	81	86
5	20–25	488	247	243	41	20	30	28
6	56–60	529	240	235	175	71	47	59
7	20–25	642	305	318	65	92	33	101
8	46–50	433	152	138	40	35	29	29
9	46–50	382	377	601	79	54	121	84
10	40–45	515	203	211	82	77	105	101
11	36–40	635	222	262	45	91	47	131
12	60–65	578	319	323	52	50	37	35
13	56–60	448	206	183	39	31	32	32
14	56–60	791	350	350	58	65	43	45
15	30–35	895	402	520	46	72	43	50
16	60–65	520	207	209	73	54	76	29
AM ± SD	49 ± 16	623 ± 188	303 ± 137	323 ± 169	68 ± 37	60 ± 21	65 ± 53	63 ± 35

*HU, Hounsfield units; ROI, region of interest; AM, arithmetic mean; SD, standard deviation; OD, oculus dexter (right optic nerve); OS, oculus sinister (left optic nerve)*.

**Table 3:** Measurements of contrast loaded cerebrospinal fluid (CLCSF) along the entire optic nerve in controls without elevated intracranial pressure and without papilledema.

**Controls**		**Basal cistern**	**Intracranial portion**		**Mid-orbital segment**		**Bulbar segment**	
		**[ROI 4]**	**[ROI 3]**		**[ROI 2]**		**[ROI 1]**	
***n***	**Age**		**OD**	**OS**	**OD**	**OS**	**OD**	**OS**
	**Years**	**HU**	**HU**		**HU**		**HU**	
1	90–95	1,067	268	230	278	151	246	242
2	40–45	607	307	312	83	100	116	166
3	46–50	881	239	206	202	180	229	141
4	30–35	595	268	299	90	84	110	149
5	30–35	807	393	331	116	115	126	146
6	66–70	500	316	308	147	109	145	127
7	76–80	368	374	327	202	200	131	131
8	66–70	527	306	350	144	188	176	168
9	46–50	860	270	335	295	289	575	529
10	70–75	391	321	299	246	202	273	185
11	76–80	610	379	359	180	185	315	208
12	46–50	639	353	284	337	234	268	266
AM ± SD	60 ± 19	654 ± 210	316 ± 50	303 ± 46	193 ± 82	170 ± 61	226 ± 131	205 ± 111

*HU, Hounsfield units; ROI, region of interest; AM, arithmetic mean; SD, standard deviation; OD, oculus dexter (right optic nerve); OS, oculus sinister (left optic nerve)*.

#### Mid-orbital segment of orbital optic nerve portion: ROI 2 (13–16 mm distant from the lamina cribrosa)

In IIH patients the mean CLCSF density measured 68 ± 37 HU in the right optic nerve and 60 ± 21 HU in the left optic nerve. In controls the mean CLCSF density measured 193 ± 82 HU in the right optic nerve and 170 ± 61 HU in the left optic nerve (Tables 2, 3).

The difference between IIH patients and controls showed statistical significance for the right and for the left optic nerve (right optic nerve: *p* < 0.001, left optic nerve: *p* < 0.001, Mann-Whitney *U*-test) (Figure 3).

#### Intracranial optic nerve portion: ROI 3 (from optic chiasm to cranial opening of the optic canal)

In IIH patients the mean CLCSF density measured 303 ± 137 HU in the right optic nerve and 323 ± 169 HU in the left optic nerve. In controls the mean CLCSF density measured 316 ± 50 HU in the right optic nerve and 303 ± 46 HU in the left optic nerve (Tables 2, 3). The difference between IIH patients and controls showed neither statistical significance for the right nor for the left optic nerve (right optic nerve: *p* = 0.75, unpaired *t-*test, left optic nerve: *p* = 0.44, Mann-Whitney *U*-test).

#### Basal cistern: ROI 4

In IIH patients the mean density of CLCSF measured 623 ± 188 HU and in controls 654 ± 210 HU (Tables 2, 3). The difference between IIH patients and controls showed no statistical significance within the basal cystern (*p* = 0.69, unpaired *t*-test).

#### CLCSF differences between ROIs

In IIH patients the mean difference between *ROI 1 and ROI 2 (ROI 1-ROI 2)* was−3 HU in the right optic nerve and 3 HU in the left optic nerve. The differences were not statistically significant (right optic nerve: *p* = 0.46, Wilcoxon Signed Rank test, left optic nerve: *p* = 0.54, paired *t*-test). The mean difference between *ROI 2 and ROI 3 (ROI 2-ROI 3)* was−235 HU in the right optic nerve and−264 HU in the left optic nerve. The differences were highly statistically significant (right optic nerve: *p* < 0.001, paired *t*-test, left optic nerve: *p* < 0.001, Wilcoxon Signed Rank test). Between *ROI 3 and ROI 4 (ROI 3-ROI 4)* the mean difference was−320 HU in the right optic nerve and−300 HU in the left optic nerve. The differences were highly statistically significant (right optic nerve: *p* < 0.001, paired *t*-test, left optic nerve: *p* < 0.001, Wilcoxon Signed Rank test). The mean difference between *ROI 1 and ROI 3 (ROI 1-ROI 3)* was−238 HU in the right optic nerve and−260 HU in the left optic nerve. The differences were highly statistically significant (right optic nerve: *p* < 0.001, paired *t*-test, left optic nerve: *p* < 0.001, Wilcoxon Signed Rank test).

In controls the mean difference between *ROI 1 and ROI 2 (ROI 1-ROI 2)* was 33 HU in the right optic nerve and 35 HU in the left optic nerve. The differences were not statistically significant (right optic nerve: *p* = 0.38, Wilcoxon Signed Rank test, left optic nerve: *p* = 0.16, paired *t-*test). The mean difference between *ROI 2 and ROI 3 (ROI 2-ROI 3)* was−123 HU in the right optic nerve and−133 HU in the left optic nerve. The differences were highly statistically significant (right optic nerve: *p* < 0.001, left optic nerve: *p* < 0.001, paired *t-*test). Between *ROI 3 and ROI 4 (ROI 3-ROI 4)* the mean difference was−338 HU in the right optic nerve and−351 HU in the left optic nerve. The differences were highly statistically significant (right optic nerve: *p* < 0.001, left optic nerve: *p* < 0.001, paired *t-*test). The mean difference between *ROI 1 and ROI 3 (ROI 1-ROI 3)* was−90 HU in the right optic nerve and−98 HU in the left optic nerve. The differences were slightly statistically significant (right optic nerve: *p* = 0.06, paired *t-*test, left optic nerve: *p* = 0.034, Wilcoxon Signed Rank test).

The difference of CLCSF density between the right and the left optic nerve was not statistically significant in either group (IIH: ROI 1: *p* = 0.96, Mann-Whitney *U*-test; ROI 2: *p* = 0.43, unpaired *t-*test; ROI 3: *p* = 0.81, Mann-Whitney *U*-test; controls: ROI 1: *p* = 0.89, Mann-Whitney *U*-test; ROI 2: *p* = 0.43, ROI 3: *p* = 0.52; unpaired *t-*test).

## Discussion

The current study uses CT cisternography to assess the CSF dynamics along the entire optic nerve in 16 patients with a history of IIH and papilledema and in 12 controls without elevated ICP and without papilledema. The measurements demonstrate a significant reduction of CLCSF concentrations within the intraorbital optic nerve segments in patients with IIH compared to controls.

The CSF pathway within the SAS of optic nerve is a closed circulatory system that ends behind the eye ball. CSF moves from the basal cistern to the intracranial optic nerve portion and then via the optic canal into the intraorbital segments (mid-orbital segment and retrobulbar segment) to the lamina cribrosa at the posterior end of the globe. Both the SAS diameter and the meshwork of trabeculae and septae that bridge the SAS differ in number and morphology within the different optic nerve portions. The narrowest diameter is measured within the intracanalicular portion where the SAS is confined by bone (7, 22–24).

Cisternography is at present the only method that can provide information on CSF flow dynamics in the SAS of the optic nerve *in vivo*. In accordance with the second law of thermodynamics a homogenous distribution of CLCSF is expected after injection of contrast agent into the CSF. The measurements of CLCSF along the entire optic nerve in the present study however clearly demonstrate a reduced CLCSF density in the orbital optic nerve segments in patients with IIH and papilledema.

The highest CLCSF density was measured in both IIH patients and controls in the basal cistern (ROI 4). This is not surprising as the volume of the chiasmal cistern is much larger than the SAS volume surrounding the optic nerve. Further, the density measured in the basal cistern is biased by the measurement method used. We measured the CLCSF as the mean of HU within a circle because CT imaging does not allow us to distinguish the exact borders of the optic nerve and the optic nerve sheath. The density measurements along the optic nerve therefore include the optic nerve sheath and the optic nerve itself which is not filled with CSF and is therefore lower than that in the basal cistern where the optic nerve density does not bias the measurement.

**Figure 4:**
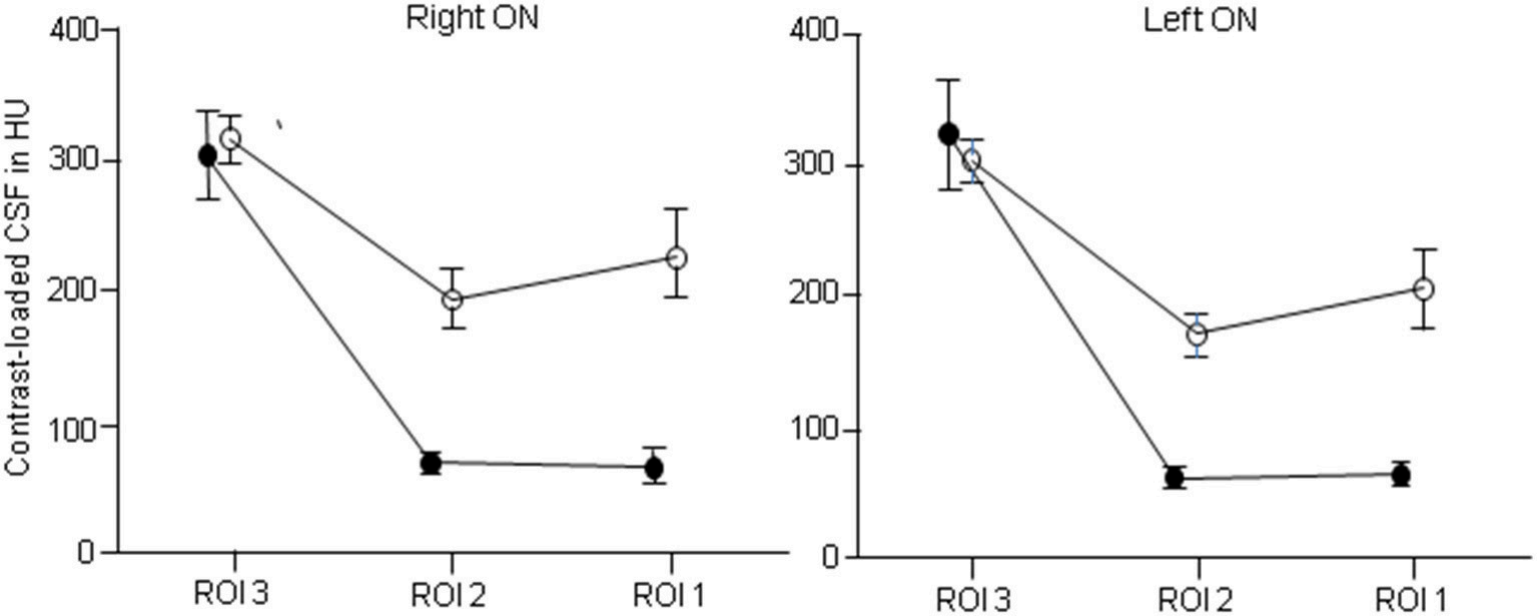
Distribution of contrast loaded cerebrospinal fluid (CLCSF) along the entire optic nerve in patients with idiopathic intracranial hypertension (IIH) and papilledema and controls, separated for right and left optic nerve. ROI 1: bulbar segment of the orbital portion, ROI 2: mid-orbital segment of the orbital portion, ROI 3: intracranial portion. •, IIH patients; ◦, controls.

**Figure 5:**
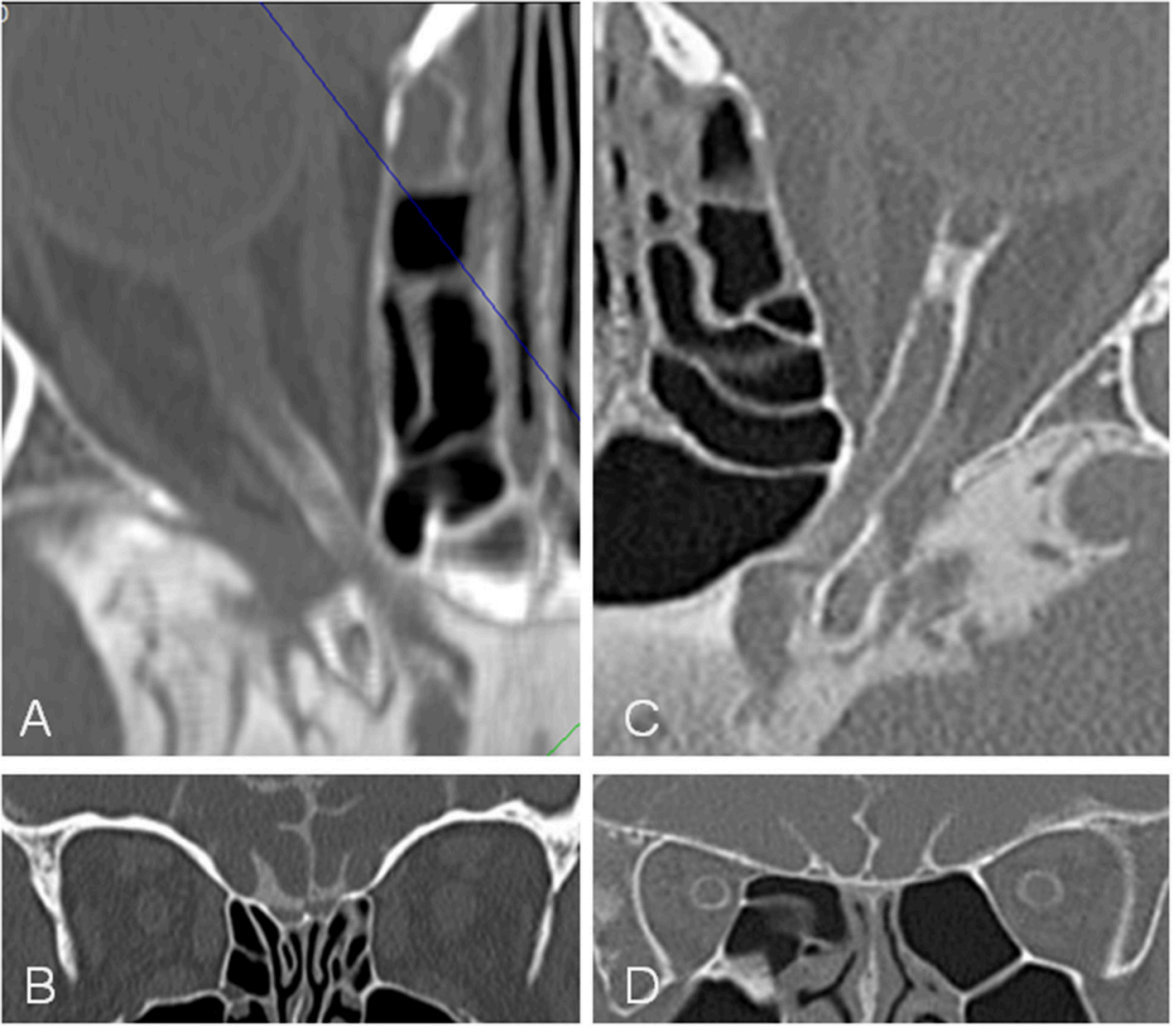
Computed tomographic (CT) cisternography in a patient with idiopathic intracranial hypertension (IIH) and papilledema and a control subject. In the patient with IIH **(A,B)** contrast loaded cerebrospinal fluid (CLCSF) inflow stops behind the optic canal while in the control subject **(C,D)** contrast loaded cerebrospinal fluid (CLCSF) flows into the orbital segment to the globe.

In both IIH patients and controls a significant reduction of CLCSF density was measured between the intracranial (ROI 3) and the mid-orbital segment of the orbital optic nerve portion (ROI 2). In controls the measured CLCSF density reduction through the optic canal is most likely to be due to the physiological diminution of the SAS diameter within the intracanalicular optic nerve portion. The SAS measured its smallest diameter within the optic canal (7, 22, 23) where the arachnoid and pia mater are merged (24). A reduced volume of CSF within the optic canal is therefore the normal condition. The gradient of CLCSF however was significantly higher in patients with IIH (right optic nerve: −235 HU, left optic nerve: −264 HU) compared to controls (right optic nerve: −123 HU, left optic nerve: −133 HU). While CLCSF density measurements were similar within the basal cistern (ROI 4) and the intracranial optic nerve portion (ROI 3) in IIH patients and controls (ROI 4: 623 HU vs. 654 HU; ROI 3: right optic nerve: 303 HU vs. 316 HU, left optic nerve = 323 HU vs. 303 HU), a highly statistically significant reduction was measured within the intraorbital optic nerve segments in patients with IIH (ROI 2: right optic nerve: 68 HU vs. 193 HU, left optic nerve: 60 HU vs. 170 HU; ROI 1: right optic nerve: 65 HU vs. 226 HU, left optic nerve: 63 HU vs. 205 HU). In controls CLCSF passes the optic canal and flows into the mid-orbital segment with a small peak in the bulbar segment behind the lamina cribrosa (ROI 1). In contrast to patients with IIH the CLCSF inflow into the orbital optic nerve SAS almost stops at the canalicular optic nerve portion and very little CLCSF reaches the bulbar segment behind the lamina cribrosa (ROI 1) (Figures 4, 5).

Evidence for the restricting effect of the optic canal on CSF dynamics in patients with IIH was provided by pro–and retrospective clinical studies (25, 26). Bidot et al. (25) investigated in a retrospective review of magnetic resonance images (MRI) the optic canal size and the severity of papilledema in 69 patients with IIH. Their measurements showed an association between the optic canal size and the severity of papilledema. More severe papilledema and poorer visual function were associated with a larger optic canal area. Accordingly, the side of less severe papilledema in 8 patients with asymmetric papilledema was associated with a smaller optic canal area (9). In a prospective study of 54 patients with intracranial pressure measurement and 600 controls (26) measured the optic canal on CT scans. This study explains why optic nerve sheath diameter monitoring may provide false-negative results in cases with narrow optic canals. In such cases there will be reduced CSF inflow into the SAS of the optic nerve causing a false negative smaller optic nerve sheath diameter.

The optic canal is confined by bone and the meninges. While bone represents the solid component, the structure of the meninges is more dynamic. The meningothelial cell (MEC) layer that forms the arachnoid and the pia layer of the meninges therefore play an important role for the anatomy of the optic canal. MECs are multifunctional cells that cover the pia and the arachnoid layer of the meninges in the entire central nervous system and thus the septae and trabeculae in the SAS of the optic nerve (22). MECs have been shown to react with growth and proliferation to mechanical stimuli such as pressure (27). Elevation of ICP in patients with IIH will therefore particularly stimulate MECs proliferation. This leads to obstruction and compartmentation of the SAS which is most crucial within the narrow SAS of the intracanalicular optic nerve portion. The “bottleneck” like characteristic of the optic canal might be particularly crucial in patients with elevated ICP and influence CSF dynamics along the optic nerve as measured in the curr]ent study. A similar finding has recently found in patients with normal tension glaucoma (28).

Such impaired CSF dynamics within the orbital optic nerve segments might be of clinical importance. Once CSF influx stagnates, the SAS of the optic nerve turns into a CSF compartment with a reduced in and outflow of CSF. This results in an accumulation of biologically active substances, such as L-PGDS, a multifactorial prostaglandin synthetase. CSF sampling during optic nerve sheath fenestration in patients with papilledema demonstrated exceedingly high concentrations of this protein (29). Recent research points to the neurotoxic effect of L-PGDS (30). Impaired CSF turnover adjacent to the optic nerve head will result in accumulation of toxic substances and reduced clearance of CSF. This may lead to neuronal, axonal and glial cell damage which may result in the clinical manifestation we observe in patients with IIH and papilledema.

Changes in the optic canal patency and formation of an optic nerve sheath compartment have also recently been considered to play a role in the development of the visual impairment intracranial pressure (VIIP) syndrome found in astronauts after prolonged space flight (31).

The present study further might be of interest for the treatment options in patients with IIH and persisting papilledema in spite of normalized ICP (12, 13). Given that an optic nerve sheath compartment has developed, optic nerve sheath fenestration that releases the local damaging pressure to the optic nerve, might be superior to a shunt procedure.

There are several obvious weaknesses in this study which need to be addressed. Firstly, the large differences in time from first diagnosis to cisternography and thus the differences in the duration of the disease in IIH patients. As cisternography is not a primary procedure in patients with IIH, cisternography was only performed in IIH patients with persistent papilledema and progressive visual impairment despite loss weight attempts and maximal therapy with systemic acetazolamide and or ventriculoperitoneal shunt surgery. The IIH patients in this study does therefore not present the “typical IIH patient.” The patients are not within the typically age range and the BMI is lower compared to the typical IIH cohort. If this is due to a cultural difference between different populations (Swiss with other countries) needs further studies. Secondly, the control group is not homogeneous and the controls underwent cisternography for various indications. Due to the invasive nature of cisternography we cannot, for obvious ethical considerations, provide a healthy aged- and gender matched control group of “normal.” However, the differences between IIH patients and the control group is clearly demonstrated. Thirdly, the density measurements in ROI 1, ROI 2, and ROI 3 are biased by including the optic nerve itself because it is still technically not possible for us to measure the CSF filled SAS alone. This however is the case in both groups and does therefore not compromise the comparison between controls and IIH. Fourthly, the number of patients in both the IIH- and the control group is rather small (IIH: *n* = 16, controls: *n* = 12) and lastly, the retrospective nature of the current study.

The current study demonstrates reduced CLCSF density within the orbital optic nerve segments in 16 (32 optic nerve) patients with history of IIH and papilledema. It is possible that impaired CSF dynamics are involved in the pathophysiology of IIH.

## Author contributions

All authors listed have contributed significantly and are in agreement with the content of the manuscript. AP was mainly involved in study design, data analysis, data interpretation and manuscript preparation. MM was mainly involved in study design, data acquisition, data interpretation and manuscript preparation. AP and MM contributed equally to this work. JP was mainly involved in data analysis. JB was mainly involved in data acquisition. LR was mainly involved in data acquisition and data analysis. HK was mainly involved in study design, data interpretation and manuscript preparation.

### Conflict of interest statement

The authors declare that the research was conducted in the absence of any commercial or financial relationships that could be construed as a potential conflict of interest.

## Abbreviations

CSF: cerebrospinal fluid
IIH: idiopathic intracranial hypertension
CT: computed tomographic
CLCSF: contrast loaded cerebrospinal fluid
HU: Hounsfield units
ROI: regions of interest
ICP: intracranial pressure
SAS: subarachnoid space
MRI: magnetic resonance imaging
BMI: body mass index
MPR: multiplanar reconstruction images
MEC: meningothelial cell
VIIP: visual impairment intracranial pressure.
